# First Nations households living on-reserve experience food insecurity: prevalence and predictors among ninety-two First Nations communities across Canada

**DOI:** 10.17269/s41997-021-00491-x

**Published:** 2021-06-28

**Authors:** Malek Batal, Hing Man Chan, Karen Fediuk, Amy Ing, Peter R. Berti, Genevieve Mercille, Tonio Sadik, Louise Johnson-Down

**Affiliations:** 1 Département de nutrition, Faculté de Médecine, Université de Montréal, Pavillon Liliane de Stewart, CP 6128 succ. Centre-Ville, Montréal, QC H3T 1A8 Canada; 2 Centre de recherche en santé publique de l’Université de Montréal et du CIUSS du Centre-sud-de-l’Île-de-Montréal (CReSP), 7101 Avenue du Parc, Montréal, QC H3N 1X7 Canada; 3 Department of Biology, University of Ottawa, 30 Marie Curie, Ottawa, ON K1N 6N5 Canada; 4 First Nations Food, Nutrition and Environment Study, University of Ottawa, 30 Marie Curie, Ottawa, ON K1N 6N5 Canada; 5 HealthBridge Foundation of Canada, 1 Nicholas Street, Suite 1004, Ottawa, ON K1N 7B7 Canada; 6 Assembly of First Nations, 55 Metcalfe Street, Suite 1600, Ottawa, ON K1P 6L5 Canada

**Keywords:** Indigenous, First Nations, Food security, Food insecurity, Food sovereignty, Traditional food, Autochtones, Premières Nations, sécurité alimentaire, insécurité alimentaire, souveraineté alimentaire, aliments traditionnels

## Abstract

**Objective:**

To describe the prevalence of food insecurity in First Nations households across Canada while identifying barriers and enablers to traditional food (TF) consumption.

**Methods:**

The First Nations Food, Nutrition and Environment Study is a cross-Canada participatory study of on-reserve First Nations from 2008 to 2018. The Household Food Security Survey Module was used to capture income-related challenges experienced by First Nations households. Households were classified as food secure, or marginally, moderately, or severely food insecure. Barriers and enablers to TF access and use were identified describing the Indigenous experience.

**Results:**

Almost half of on-reserve First Nations households were food insecure and the prevalence was higher than that for non-Indigenous households in Canada. On-reserve food insecurity prevalence was higher in western regions of Canada. First Nations households with children experienced greater food insecurity than those without children. More adults experienced severe food insecurity than children. Most adults would like to have more TF in their diet but state that factors such as financial and household constraints, industrial activities, government regulations, climate change, and fear of contamination impede greater access. Food costs were substantially higher in remote First Nations communities, but remoteness was not associated with food security in multivariable analysis.

**Conclusion:**

Existing systems have been unsuccessful in curbing the food insecurity in First Nations households. Improving food security hinges on achieving Indigenous Food Sovereignty, the key to long-term conservation and stewardship of the land and the co-management of these by Indigenous Peoples. Studies investigating the feasibility of increasing TF from an Indigenous perspective are required.

## Résumé

**Objectifs:**

Décrire la prévalence de l’insécurité alimentaire chez les ménages des Premières Nations du Canada tout en identifiant les obstacles et les facilitateurs de l’alimentation traditionnelle (AT).

**Méthodes:**

L’étude sur l’alimentation, la nutrition et l’environnement des Premières Nations dans les réserves est une étude participative pancanadienne des ménages des Premières Nations. Le module d’enquête sur la sécurité alimentaire des ménages a été utilisé pour tenir compte des défis liés au revenu qui confrontent les ménages des Premières Nations. Les ménages ont été classés comme étant en sécurité alimentaire ou insécurité alimentaire marginale, modérée ou sévère. Des obstacles et facilitateurs à l’accès et à l’utilisation des AT ont été identifiés pour décrire l’expérience autochtone.

**Résultats:**

Près de la moitié des ménages dans les réserves des Premières Nations dans notre étude étaient en situation d’insécurité alimentaire et la prévalence était supérieure aux ménages non-Autochtones du Canada. L’insécurité alimentaire dans les réserves était plus élevée dans les régions de l’Ouest. Les ménages des Premières Nations ayant des enfants ont connu une plus grande insécurité alimentaire que ceux qui n’en ont pas. Plus d’adultes que d’enfants ont connu une insécurité alimentaire sévère. La plupart des adultes aimeraient avoir plus d’AT dans leur alimentation, mais affirment que des facteurs tels que les contraintes financières et domestiques, les activités industrielles, les réglementations gouvernementales, les changements climatiques et la peur de la contamination empêchent un meilleur accès. Les coûts des aliments étaient considérablement plus élevés dans les communautés isolées des Premières Nations, mais l’isolement n’était pas associé à la sécurité alimentaire dans l’analyse multivariée.

**Conclusion:**

Les systèmes existants ont échoué à freiner l’insécurité alimentaire chez les ménages des Premières Nations. L’amélioration de la sécurité alimentaire dépend de la réalisation de la souveraineté alimentaire autochtone, une clé pour la conservation à long terme des ressources naturelles et pour une gestion partagée de ces ressources par les peuples autochtones. Des études menées dans une perspective autochtone sur la faisabilité d’améliorer l’accès aux AT sont encore nécessaires.

## Introduction

Food security is achieved “... when all people, at all times, have physical, social and economic access to sufficient, safe and nutritious food to meet their dietary needs and food preferences for an active and healthy life” (Food and Agriculture Organization 2008). Most household food security measures assess the inadequate or insecure access to store-bought foods as a result of financial constraints. A common tool to measure food security in Canada is the Household Food Security Survey Module (HFSSM) used in the national Canadian Community Health Survey (CCHS) (Health Canada 2007; PROOF Food Insecurity Policy Research 2018).

Because food security is largely looked at through the lens of economics and physical access, broader concepts are required to capture the distinct food systems of Indigenous Peoples and to honour their unique world views and perspectives. Stated as a precondition for food security, Indigenous Food Sovereignty emphasizes the close connection Indigenous Peoples have with their environment, the work being done by Indigenous communities to revitalize their food systems, the transmission of cultural knowledge about their lands, and the harvesting of traditional food (TF) (i.e., hunting, fishing, gathering, cultivating) (Coté 2016; Indigenous Peoples in Route to the Rio +20 Conference 2011). This concept is often used to exemplify the struggles First Nations Peoples encounter in harvesting TF and restoring/revitalizing their Indigenous food systems (Morrison 2006, 2011).

Food security and food sovereignty are inexorably linked for Indigenous Peoples because the harvest of TF is a key element in the achievement of both these constructs (Centre for Indigenous Conservation and Development Alternatives 2017; Morrison 2006, 2011). Much of the difficulties in harvesting TF stem from the ongoing long-standing colonial policies that have led to a loss of governance over Indigenous lands, and the breakdown of social systems from the intergenerational trauma of the residential school system that saw the removal of children from their families and other institutions that separated them from their culture. Land privatization, government regulations, and overexploitation of resources from industrialization (e.g., hydro-electricity, mining, forestry, and roadways) along with climate change have led to substantive environmental degradation, including increased contaminant risk from pollution, biodiversity losses, and the inability to access sufficient amounts of TF (Coté 2016; Laberge Gaudin et al. 2015; Turner et al. 2013b). Other inhibiting factors include the time and resources needed to access distant harvest areas, changes in knowledge and skills related to harvesting and preparation of TF, and avoidance of areas and food due to concerns for contaminants (Chan et al. 2006; Delormier and Marquis 2018; Kuhnlein et al. 2013; Laberge Gaudin et al. 2015). The multiple threats impacting First Nations Peoples’ ability to maintain reliance on the TF systems combined with inadequate support from existing health systems are accompanied by the socio-demographic risk factors for food insecurity (Tarasuk et al. 2016, 2019).

A higher proportion of First Nations individuals live in poverty as compared with other Canadians and they more often live in poorly constructed, over-crowded houses headed by single parents (Statistics Canada 2015; Tarasuk et al. 2016). Using CCHS data, Tarasuk et al. (2019) found that the probability and severity of food insecurity are more than 50% (or between 34% and 78%) higher in off-reserve Indigenous households than in the general population of Canada. The First Nations Regional Health Survey reported that food insecurity was experienced by 54% of First Nations individuals living on-reserve (First Nations Information Governance Centre 2012).

Food insecurity is linked to inadequate intakes of several nutrients, the inadequacy of which could explain in part the health inequities experienced by Indigenous populations (Li et al. 2016; Willows 2005). First Nations Peoples exhibit a high prevalence of nutrition-related chronic disease (NRCD) such as obesity and type 2 diabetes (Public Health Agency of Canada 2011a, b). Prevalence of obesity in First Nations Peoples is reaching alarming levels at 50%, and type 2 diabetes is rampant with a prevalence of 19%, both double that of other Canadians (Batal et al. 2021b).

With the aim to offset food insecurity and because food costs are more expensive in remote Indigenous communities, some programs have been instituted, among them ‘Nutrition North’ since 2011, a program managed by the government of Canada that provides subsidies for a variety of perishable and nutritious food items and commercially processed TF to remote Indigenous communities (Nutrition North Canada 2018). Regrettably, this program has not managed to effectively reduce food insecurity as remote fly-in First Nations and Inuit communities that are eligible for this program continue to experience the highest prevalence of food insecurity in Canada (Tarasuk et al. 2019). On the other hand, First Nations individuals have managed to survive and sometimes thrive despite these challenges with community-based food security programming (Skinner et al. 2012), particularly with land-based food procurement strategies (i.e., strategies that improve access to TF through the support of TF gathering activities and the sharing of resources) (Leibovitch Randazzo and Robidoux 2019; Thompson et al. 2014).

Because food security in an Indigenous context cannot be separated from issues of food sovereignty and access to the preferred TF, in this article, in addition to reporting food security prevalence and analyzing factors contributing to it, we will investigate drivers of food sovereignty by identifying barriers and enablers of TF as expressed by on-reserve First Nations adults across Canada.

## Methods

The First Nations Food, Nutrition and Environment Study (FNFNES) is a cross-Canada participatory study of First Nations adults living on-reserve south of the 60^th^ parallel. Recruitment and sampling occurred from 2008 to 2016 and these have been described elsewhere (Chan et al. 2021). Data collection began in British Columbia in 2008–2009, followed by Manitoba (2010), Ontario (2011–2012), Alberta (2013), the Atlantic region (New Brunswick, Nova Scotia, Prince Edward Island and Newfoundland (excluding Labrador)) (2014), Saskatchewan (2015), and Quebec/Labrador (2016). Methods were presented for approval to participating First Nations communities in each region before the onset of data collection in a regional methodology workshop and adjustments were made to the methods to respond to communities’ needs. The required adjustments did not affect the survey tools used to assess food security and TF access, both discussed in the present article. As a result, the same tools were used in the 92 participating communities. Community-level results were presented to each participating First Nations community’s leadership and members, and reactions were obtained to the findings for each section of the study and incorporated in the final community report. Anonymized regional results were subsequently presented at regional workshops to obtain reactions from regional First Nations representatives before any results were published. Each Nation’s data were returned to the First Nation’s leaders and members and community representatives were trained in data analysis.

Interviews to assess food security were conducted by trained First Nations workers under the guidance of a trained dietitian using the HFSSM. The HFSSM module focuses on self-reports of uncertain, insufficient, or inadequate food access, availability and utilization due to limited financial resources, and the compromised eating patterns and food consumption that may result among members of a household (Health Canada 2012; PROOF Food Insecurity Policy Research 2018). The questionnaire contained 18 questions (10 questions for adults and 8 additional questions for households with children) (PROOF Food Insecurity Policy Research 2018). Households were classified as food secure if no item was answered positively, or as marginally, moderately, or severely food insecure, using the thresholds adopted by PROOF (PROOF Food Insecurity Policy Research 2018).

In the third year of the FNFNES (after data collection was completed in British Columbia), food costs in participating First Nations communities and in one or more major urban centres in each region were estimated using the National Nutritious Food Basket tool (Health Canada 2009). The total costs of the items in the food basket as recorded by the field research staff were used to calculate the weekly cost of a food basket for a family of four consisting of two adults (a woman and a man, aged 31–50 years) and two children (one boy aged 14–18 years and one girl aged 4–8 years).

Prevalence of food insecurity was compared geographically by region (British Columbia, Alberta, Saskatchewan, Manitoba, Ontario, the Atlantic region, and Quebec and Labrador). Food insecurity was also compared by the Indigenous and Northern Affairs Canada Remoteness Index Zone (INACRIZ) classification (Alasia et al. 2017): Zone 1 (year-round road access and within 50 km to the nearest service centre); Zone 2 (year-round road access and between 50 and 350 km to the nearest service centre); Zone 3 (year-round road access and >350 km to the nearest service centre); Zone 4 (no year-round road access to a service centre, i.e., fly-in First Nations communities) (Alasia et al. 2017).

Other information collected by interviewers included information on diet, lifestyle, environmental concerns, and health. Information on household socio-demographic characteristics (gender, age group, income source, number of adults with full-time employment, education) and health (self-reported health, BMI, smoking status) were also collected. Although income was not collected, education and source of income were used as proxies. Because the HFSSM does not measure TF access, FNFNES measured indicators related to Indigenous Food Sovereignty using additional questions that addressed the barriers and enablers to obtaining TF and the control over access and sufficiency of TF. Two questions (“We worried whether our traditional food would run out before we could get more” and “The traditional food that we got just didn’t last, and we couldn’t get any more.”) were posed to assess a household’s adequacy of and the ability to replenish TF supplies. Additional questions, some open-ended, investigating perceptions of the impact of climate change on TF consumption and barriers to greater use of TF, plus another close-ended question listing possible impediments to TF harvesting, were administered. Answers to the open-ended questions were categorized by an analyst into at least one of the main themes that emerged (financial, household, personal constraints, climate change, etc.) and analyzed as a percentage of mentions of each theme over the total number of responses.

Food insecurity prevalence was the dependent variable in a multivariable logistic regression. Individuals with ‘severe’, ‘moderate’, or ‘marginal’ food insecurity were grouped together and compared to food secure individuals. The independent variables included the region in which the respondent resided; whether the First Nations community had year-round road access; the number of individuals in the house with full-time work (0, 1, or 2 or more); the main source of income (wages, salary, or self-employment vs. all other sources); age group (19–30, 31–50, 51–70, 71 years or over); the individual’s BMI category (less than 25, 25 to less than 30, or 30 and above); the individual’s formal education level (8 years or less, 9 to 12 years, 13 years or more); gender; and self-reported health (good (combined ‘excellent’, ‘very good’ and ‘good’) and poor (combined ‘fair’ and ‘poor’)).

Data were entered into a study database using Epi Info 3.5.4 (Centers for Disease Control and Prevention, Atlanta, GA, USA). Data analysis used SAS/STAT version 9.4 (SAS, Cary, NC, USA, 2013). All analyses were weighted by the First Nations community, household, and individual for non-response and to ensure they represented this population. Weights were adjusted for changes in population from 2008 to 2017. For the multivariable analysis, replicate weights were generated by randomly selecting 500 bootstrap subsamples of the full sample. Using PROC SURVEYLOGISTIC in SAS, the balanced repeated replication (BRR) option for variance estimation was employed (‘VARMETHOD=BRR’).

## Results

**Fig. 1:**
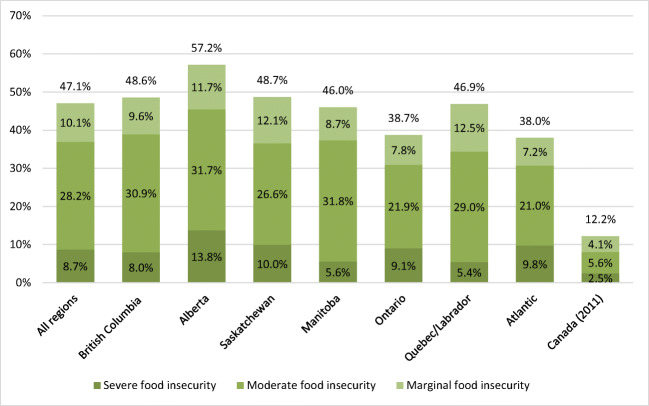
Household food insecurity prevalence by region for First Nations households in Canada from the First Nations Food, Nutrition and Environment Study (FNFNES), 2008–2018, compared with the previously reported national prevalence for Canada. National food insecurity rate for Canada presented here for comparison taken from *Household food insecurity in Canada, 2012* (Tarasuk et al. 2014). The researchers calculated rates from CCHS data gathered in 2011 among Canadian households. Household food insecurity among Indigenous households located off-reserve was 27.1%

The final sample size was 5176 after removing all missing data in the multivariable analysis. Almost half (47.1%) of all participating households were food insecure, while regional prevalence ranged from 38.0% to 57.2% (Fig. 1). Household food insecurity prevalence was higher in British Columbia and Alberta as compared with that in the Atlantic region.

First Nations household food insecurity prevalence among households with and without children is presented in Table 1. Forty-eight to 74% of households had children under the age of 18 years depending on the region. First Nations households with children experienced greater food insecurity than those without children (54% vs. 36%) and 29% of these households reported food insecurity at the child level, i.e., the children themselves experienced food insecurity (Table 1). In most regions, more than 5% of households with children exhibited severe food insecurity. The prevalence of food insecurity in households without children in Alberta was higher when compared with the Atlantic region, Ontario, and Saskatchewan but not different from British Columbia, Manitoba, and Quebec/Labrador. More adults in households with children experienced severe food insecurity than children (9% of adults with severe food insecurity vs. 3% of children) (Table 1).

**Table 1:** Income-related household food security status for First Nations households with and without children in Canada from the First Nations Food, Nutrition and Environment Study (FNFNES), 2008–2018^a^

		Food secure		Food insecure							
		All		All		Marginal		Moderate		Severe	
		*n*	% (95% CI)	*n*	% (95% CI)	*n*	% (95% CI)	*n*	% (95% CI)	*n*	% (95% CI)
All households	Household status	2878	52.9 (48.5–57.4)	2298	47.1 (42.6–51.5)	489	10.1 (9.0–11.1)	1328	28.2 (24.5–31.7)	481	8.7 (7.0–10.5)
	Adult status	2969	54.7 (50.2–59.2)	2170	44.7 (40.3–49.1)	414	8.6 (7.6–9.7)	1285	27.7 (24.4–31.0)	471	8.4 (6.5–10.2)
	Child status	1832	62.3 (56.3–68.3)	865	28.9 (24.3–33.6)	147	3.3 (2.3–4.3)	644	20.9 (17.3–24.5)	74	3.1 (2.0–4.2)
Households with children	Household status	1469	47.7 (44.0–48.0)	1489	52.3 (46.7–57.9)	332	11.9 (10.0–13.7)	876	31.1 (26.5–35.6)	281	9.4 (7.1–11.6)
	Adult status	1560	50.4 (44.7–56.0)	1361	48.8 (43.3–54.3)	257	9.7 (7.9–11.5)	833	30.2 (25.9–34.6)	271	8.9 (6.5–11.2)
	Child status	1832	62.3 (56.3–68.3)	865	28.9 (24.3–33.6)	147	4.9 (4.5–6.3)	644	20.9 (17.3–24.5)	74	3.1 (2.0–4.2)
Households without children	Household status	1409	63.8 (60.1–67.4)	809	36.2 (32.5–39.9)	157	6.4 (4.9–7.8)	452	22.4 (19.2–25.6)	200	7.4 (5.5–9.3)

*CI*, confidence interval

^a^Frequencies and confidence intervals are weighted

**Fig. 2:**
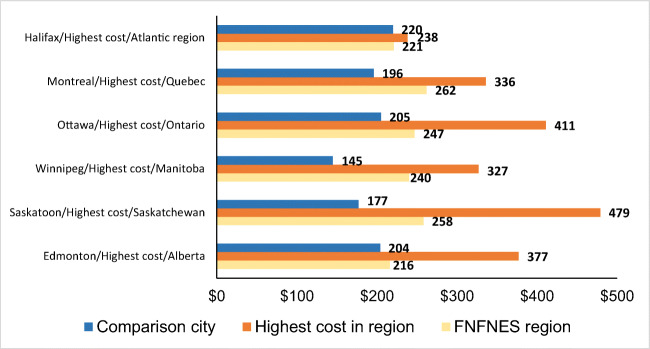
Comparison of average cost of healthy food basket to the community with the highest cost and a main urban centre in each region. Food basket costing was not undertaken in BC

In all regions, food costs were lower in major urban cities (Fig. 2). Cost differences between the urban centre and First Nations communities were lowest in the Atlantic region. Weekly food basket costs were $112–$140 higher in remote First Nations communities without road access vs. non-remote communities.

**Fig. 3:**
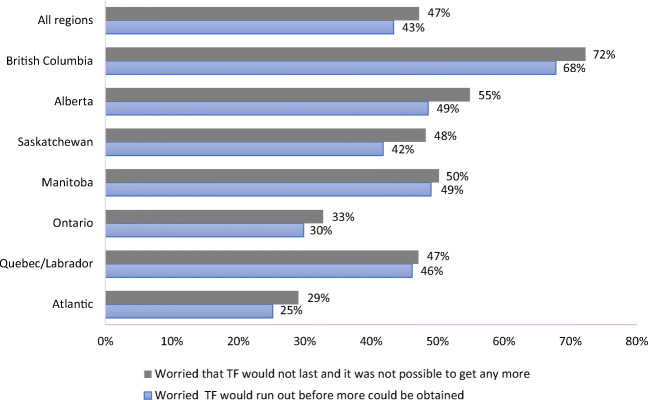
The percentage of First Nations households who experienced a traditional food shortage and worried about the status of their traditional food supply in the last 12 months in Canada from the FNFNES, 2008–2018

Most adults said that they would like to have more TF in their diet. Although two thirds of First Nations households were actively engaged in harvesting (67%), over half of all adults reported that industrial activities (i.e., mining, hydro-electricity, forestry, farming) affected their harvesting in addition to other factors including climate change, financial (money and equipment or transportation), and household constraints (no hunter, time, traditional knowledge, and health). Overall, 47% indicated that they had experienced TF shortages while 43% were concerned that they could not get more in a timely fashion (Fig. 3).

Results of multivariable analyses are summarized in Table 2. The Atlantic region reported less food insecurity than Alberta and British Columbia. Women vs. men, households with children vs. those without, and individuals on social assistance, workers’ compensation, and employment insurance vs. those receiving wages reported greater food insecurity. Participants with poor perceived health vs. those reporting good health and smokers vs. non-smokers reported food insecurity. Those households reporting TF activities were more food insecure than those not reporting it. Age, body mass index, and food basket cost did not influence prevalence of food insecurity.

**Table 2:** Multivariable predictors of food insecurity among First Nations households in Canada from the FNFNES, 2008–2018

		Household food insecurity Percent (95% CI)	Adjusted odds ratio (95% CI)	*p* value
Region	British Columbia	48.6 (41.9, 55.3)	1.91 (1.02, 3.56)	0.01
	Alberta	57.2 (43.3, 71.1)	1.66 (1.11, 2.46)	0.04
	Saskatchewan	48.7 (40.5, 56.8)	1.28 (0.84, 1.97)	0.25
	Manitoba	46.0 (34.5, 57.6)	1.10 (0.55, 2.21)	0.79
	Ontario	38.7 (28.3, 49.2)	1.12 (0.65, 1.92)	0.68
	Quebec/Labrador	46.9 (27.3, 66.5)	1.31 (0.76, 2.28)	0.33
	Atlantic region	38.0 (34.6, 41.3)	Ref.	
Year-round road access	No	57.5 (49.7, 65.3)	1.74 (0.55, 5.52)	0.34
	Yes	46.1 (41.4, 50.8)	Ref.	
Number of full-time workers in the home	0	57.0 (50.8, 63.3)	1.64 (1.11, 2.44)	0.01
	1	44.7 (38.8, 50.6)	1.40 (0.96, 2.05)	0.08
	2 or more	36.6 (31.0, 42.2)	Ref.	
Household traditional food activities	Yes	48.9 (44.6, 53.2)	1.57 (1.24, 1.97)	0.00
	No	43.2 (37.7, 48.7)	Ref.	
Source of income	Wages	37.6 (33.9, 41.2)	Ref	
	Social assistance	64.9 (56.6, 73.2)	2.49 (1.81, 3.43)	0.00
	Pension	39.7 (33.6, 45.7)	1.21 (0.81, 1.82)	0.35
	Workers’ compensation/employment insurance	58.3 (44.9, 71.7)	2.00 (1.07, 3.72)	0.03
	Other	52.4 (34.8, 69.9)	1.59 (0.85, 2.99)	0.15
Age group	19–30 years	48.6 (40.3, 56.8)	0.94 (0.43, 2.06)	0.89
	31–50 years	50.4 (45.7, 55.2)	1.17 (0.61, 2.24)	0.64
	51–70 years	41.9 (37.3, 46.6)	0.91 (0.49, 1.71)	0.78
	71 years and over	39.8 (28.1, 51.4)	Ref.	
Body mass index	< 25	49.5 (43.9, 55.1)	Ref.	
	25– < 30	44.3 (38.3, 50.2)	0.96 (0.71, 1.30)	0.81
	30 and over	48.1 (43.3, 52.8)	1.13 (0.90, 1.42)	0.28
Years of education	8 or less	52.2 (45.2, 59.1)	1.39 (0.96, 2.00)	0.08
	9 to 12	48.6 (43.5, 53.8)	1.23 (0.91, 1.65)	0.18
	13 or more	36.6 (29.5, 43.8)	Ref.	
Gender	Women	50.0 (44.7, 55.2)	1.46 (1.12, 1.90)	0.00
	Men	41.4 (36.5, 46.3)	Ref.	
Smoking	No	41.0 (35.9, 46.1)	0.75 (0.59, 0.97)	0.03
	Yes	52.6 (47.7, 57.6)	Ref.	
Health perception	Poor	54.3 (49.1, 59.6)	Ref.	
	Good	45.5 (40.8, 50.1)	0.71 (0.57, 0.87)	0.00
	Very good to excellent	40.5 (33.7, 47.3)	0.63 (0.46, 0.86)	0.00
Households with children	Yes	52.3 (46.7, 57.9 )	1.79 (1.41, 2.27)	0.00
	No	62.2 (32.5, 39.9)	Ref.	
Food basket cost			1.00 (0.99, 1.01)	0.93

*CI*, confidence interval; *Ref*, reference category

## Discussion

For millennia, Indigenous Peoples have taken care of the land, cultivating, producing, and harvesting foods in sustainable ways because of their “long-standing sacred responsibilities to nurture healthy, interdependent relationships with the land, plants, and animals that provide us with our food” (Morrison 2011). Tragically, the legacy of colonialism and privatization has resulted in the widespread environmental dispossession of Indigenous Peoples’ lands and their ability to fully maintain this relationship (Kuhnlein and Receveur 1996; Turner et al. 2013a; Whyte 2015). More recently, extreme weather fluctuations created by climate change have emerged to further complicate the adaptations required to maintain Indigenous Food Systems (Ford 2012).

As a result of the challenges facing them, there continues to be an inequitable distribution of the economic resources and other social determinants of health between First Nations Peoples and others in Canada (Egeland and Harrison 2013; Turner et al. 2013a; United Nations 2007; Whyte 2015). Policy decisions impacting environmental determinants of health and well-being are often made for the benefit of external entities to First Nations communities, such as for industrial development and other territorial considerations that often do not consider First Nations right to self-determination (Turner et al. 2013a; Whyte 2015). The outcome is a dire situation where a majority of First Nations households are not able to achieve a healthy diet either from the TF systems within their territory due to these external considerations now compounded by the climate crisis, or from the market food system due to financial constraints that limit access to diverse and high-quality market foods (Kuhnlein et al. 2009; Lambden et al. 2006; Willows et al. 2019). As a result, income-related food insecurity prevalence is four times higher among First Nations households on-reserve than among non-Indigenous Canadian households (48% vs. 12%) and greater than that among off-reserve Indigenous households (27%) (Tarasuk et al. 2019).

When household TF activities were reported, they were associated with higher food insecurity, indicating that the costs associated with these activities (owning and the use of equipment) (Pal et al. 2013) may contribute to an increased food insecurity or, alternatively, these foods were resorted to in case of food insecurity (Schuster et al. 2011). Our cross-sectional data cannot establish, however, the reason for the observed association; careful qualitative research is needed to better understand the relationship between food security and TF harvesting.

Household food security has previously been associated with income-related issues such as low revenue and lower formal education attainment (Tarasuk et al. 2016). FNFNES measured education level and income source of respondent (i.e., employment and social assistance), along with employment status of household members but not individual or household income level. Although First Nations households located off-reserve with lower levels of formal education report four times higher risk of food insecurity (Tarasuk et al. 2019), FNFNES found no such reduction of risk in on-reserve First Nations households who reported higher education level. We observed that a combination of insufficient employment at the household level (less than 2 adults working full-time) and the respondent’s source of income appeared to be income-related contributing factors to the high levels of food insecurity in on-reserve First Nations households.

As in other studies (Egeland et al. 2010; Tarasuk et al. 2016; Willows et al. 2011), our results clearly showed that households with children experience higher prevalence of food insecurity than those without children. As with other studies, to mitigate this, children in FNFNES households tended to be protected from food insecurity, and particularly so from its most severe form, by the adults sacrificing their food security on behalf of their offspring (9% of adults with severe food insecurity vs. 3% of children) (Power 2008; Tarasuk et al. 2016). In contrast, earlier studies in Indigenous Peoples in Canada report that children’s reported food insecurity is worse than that of adults (Power 2008). It is possible, however, that some severe food insecurity figures were underestimated in this study, particularly in households with children; several community representatives mentioned, during the return of community results, that some parents, for fear of being stigmatized or having social workers interfere with their raising of their children, might be underreporting food insecurity (personal correspondence to the first author).

Unlike in Tarasuk et al. (2019), the probability of household food insecurity was higher for First Nations Peoples in Alberta than for those in the Atlantic region. The high levels of income-related food insecurity across most regions and the challenges of including more TF in the diet largely explain the less desirable dietary patterns (characterized by low intakes of grains, dairy, fruits and vegetables, and high intakes of sugar and sources of saturated fat and salt such as processed meats) and inadequate intake of several nutrients that we have found previously (Batal et al. 2018; Batal et al. 2021a; Willows et al. 2019). Household financial constraints play a key role in the quality and quantity of the household food supplies and dietary intake (Tarasuk et al. 2007). With a limited food budget, many First Nations families rely on lower priced foods which are often lower in nutritional value and are ultra-processed (Batal et al. 2018).

Food insecurity is associated with a higher likelihood of obesity and other NRCD such as diabetes, and the First Nations Regional Health Survey reports similar results to ours (First Nations Information Governance Centre 2018; Vozoris and Tarasuk 2003). Without strong efforts to decrease the prevalence of food insecurity, it will be difficult to address the prevalence of obesity and NRCD among First Nations Peoples—the very problems caused by food insecurity (Galesloot et al. 2012; Vozoris and Tarasuk 2003). Furthermore, Willows et al. (2012) affirm that food security is important for obesity prevention specifically in First Nations Peoples. It is important to note that those participants with poor health perception reported lower prevalence of food security (OR 1.39).

The strengths of this study include measuring food security using the HFSSM validated tool (PROOF Food Insecurity Policy Research 2018) and having a large sample with weighting of results at the First Nations community and regional levels, while accounting for population growth within these First Nations communities. Because the diet of First Nations individuals is a mix of both TF and store-bought food and the HFSSM only captures the latter, additional questions explored the barriers and concerns related to the harvesting, sharing, preparation, and consumption of TF, as TF practices are central to First Nations culture, health, and survival (Power 2008). Bias may have been introduced due to self-reported data that tend to over-report items viewed as positive such as the increased consumption of TF and under-report items interpreted as negative such as the intake of unhealthy market foods (Skinner et al. 2013).

Another strength was that this study provided new insight into the large variation in food costs between urban and rural First Nations communities. Food cost estimates, however, may underestimate the true costs of food as they did not include transportation costs involved in obtaining the food. In our study, food costs were restricted to items in the Healthy Food Basket (Health Canada 2009), a tool with a limited amount of foods, developed to help comparisons across regions but not estimating the real cost of foods consumed by First Nations Peoples. This basket only contains market foods and may not reflect foods culturally important to First Nations individuals. Interestingly, after adjusting for other factors, First Nations community food costs were not associated with food insecurity in our data.

Like Tarasuk et al. (2016), we included the marginally food insecure households in our analyses (those who are worried about having enough money to buy food). Because of this approach, our estimates would be higher than those reported by Statistics Canada and Health Canada as they only include the moderate and severe classifications in food insecurity (Health Canada 2017; Statistics Canada 2018). Severely food insecure households experience regular disruptions to eating patterns and food shortages, and moderately food insecure may be purchasing lower quality foods (Tarasuk et al. 2016).

Despite the introduction of the ‘Nutrition North Canada’ subsidy program since 2011 (Nutrition North Canada 2018), a high prevalence of food insecurity remains entrenched in remote Indigenous communities in Canada. Measured food insecurity in Nunavut at 41% (north of the 60^th^ parallel) was the highest in Canada in 2014 (Tarasuk et al. 2016, 2019), similar to the prevalence in remote First Nations communities in our sample south of the 60^th^ parallel at 43–58%. The Office of the Auditor General report in 2014 identified three main problems with ‘Nutrition North’ that may explain its failure to abate food insecurity in remote Indigenous communities: Indigenous community eligibility was not based on need; it is unclear whether subsidies were passed on to consumers; and insufficient information was collected to manage the program or monitor its success (Auditor General of Canada 2014).

First Nations Peoples are seeking greater control over their food systems through self-determination in order to achieve better food sovereignty (Coté 2016; Kepkiewicz and Dale 2019). Governments need to continue to encourage and build upon current First Nations Peoples efforts at all levels (i.e., First Nations community, regional, provincial, and national) to improve the food security and sovereignty of First Nations Peoples and provide adequate funding for programs developed by First Nations to improve access to TF (Solar and Irwin 2010). Future research/monitoring efforts need to supplement data at the individual level with higher level environmental data for both the market and Indigenous Food Systems (including market food availability, access, pricing, marketing, the ability of First Nations Peoples to influence food grown and sold within the community, TF access, distribution channels, and evaluation of success of interventions).

New mechanisms and governance models, co-developed with First Nations Peoples, are needed to address weaknesses in current policy and program approaches. This may range from increasing local food production and reducing food price differences to improving families’ financial ability to obtain healthy food choices. Additionally, to address environmental threats to TF systems, efforts are needed that strengthen First Nations Peoples’ self-determination and connection to the land, and their stewardship of the environment (Lee et al. 2019). Indigenous priorities and values need to be meaningfully recognized and included within relevant federal, provincial, and municipal decisions with respect to land use, development, conservation, and habitat protection, with an intention to maintain or enhance access to and availability of high-quality TF, as has been recognized in the United Nations Declaration on the Rights of Indigenous Peoples (United Nations 2007). Support is needed by all levels of government, including First Nations Peoples themselves, to monitor, protect, and ensure that local ecosystems are healthy and can support First Nations Peoples’ ability to access TF.

Some First Nations communities have allied to implement programs to improve their food sovereignty, such as reintroducing and promoting salmon in the Okanagan (Syilx Okanagan Nation Alliance 2017), paying individuals who hunt and fish full-time (Cree Hunters and Trappers Income Security Board 2017), and championing ongoing efforts for knowledge transfer to future generations, e.g., culture camps and school curricula. Studies investigating the holistic evaluation of the feasibility of increasing traditional foods from an Indigenous perspective are required to monitor the health, social, and economic benefits of TF and food sovereignty strategies.

## Data Availability

Data are owned by each participating community. The Assembly of First Nations is data custodian and any requests will be addressed to AFN through the corresponding author.

## Code availability

Analysis codes can be shared upon reasonable request.
